# Genetic Mechanism of Human Neutrophil Antigen 2 Deficiency and Expression Variations

**DOI:** 10.1371/journal.pgen.1005255

**Published:** 2015-05-29

**Authors:** Yunfang Li, David C. Mair, Randy M. Schuller, Ling Li, Jianming Wu

**Affiliations:** 1 Department of Veterinary and Biomedical Sciences, University of Minnesota, Saint Paul, Minnesota, United States of America; 2 American Red Cross, North Central Blood Services, National Neutrophil Reference Laboratory, Saint Paul, Minnesota, United States of America; 3 Department of Clinical and Experimental Pharmacology, University of Minnesota, Minneapolis, Minnesota, United States of America; 4 Department of Medicine, University of Minnesota, Minneapolis, Minnesota, United States of America; University of Washington, UNITED STATES

## Abstract

Human neutrophil antigen 2 (HNA-2) deficiency is a common phenotype as 3–5% humans do not express HNA-2. HNA-2 is coded by *CD177* gene that associates with human myeloproliferative disorders. HNA-2 deficient individuals are prone to produce HNA-2 alloantibodies that cause a number of disorders including transfusion-related acute lung injury and immune neutropenia. In addition, the percentages of HNA-2 positive neutrophils vary significantly among individuals and HNA-2 expression variations play a role in human diseases such as myelodysplastic syndrome, chronic myelogenous leukemia, and gastric cancer. The underlying genetic mechanism of HNA-2 deficiency and expression variations has remained a mystery. In this study, we identified a novel *CD177* nonsense single nucleotide polymorphism (SNP 829A>T) that creates a stop codon within the *CD177* coding region. We found that all 829TT homozygous individuals were HNA-2 deficient. In addition, the SNP 829A>T genotypes were significantly associated with the percentage of HNA-2 positive neutrophils. Transfection experiments confirmed that HNA-2 expression was absent on cells expressing the *CD177* SNP 829T allele. Our data clearly demonstrate that the *CD177* SNP 829A>T is the primary genetic determinant for HNA-2 deficiency and expression variations. The mechanistic delineation of HNA-2 genetics will enable the development of genetic tests for diagnosis and prognosis of HNA-2-related human diseases.

## Author Summary

Human neutrophil antigen 2 (HNA-2) is coded by *CD177* gene that involves in human myeloproliferative disorders. HNA-2 expression varies among humans and about 3–5% people lack HNA-2 expression. HNA-2 deficient people are susceptible to produce HNA-2 alloantibodies, which play a pathological role in various human diseases including transfusion-related acute lung injury, neonatal alloimmune neutropenia, autoimmune neutropenia, drug-induced immune neutropenia, and graft failure following marrow transplantation. The level of HNA-2 expression has also been identified as a prognostic biomarker for the gastric cancer. Although HNA-2 is among the most important clinical antigens, the underlying genetic mechanism of HNA-2 deficiency and expression variations has remained unknown. Here, we demonstrate that HNA-2 deficiency and expression variations are primarily caused by a novel *CD177* genetic polymorphism that disrupts HNA-2 expression. The illumination of genetic mechanism for HNA-2 deficiency and expression variations will enable the development of effective HNA-2 genetic tests. Our findings will facilitate prognosis and diagnosis of HNA-2-related human disorders.

## Introduction

Transfusion-related acute lung injury (TRALI) is associated with the transfusion of leukocyte alloantibodies from donors or associated with the presence of alloantibodies in recipients of blood [1,2]. Alloantibodies against human neutrophil alloantigenes (HNAs) are a very strong trigger for the development of TRALI [1,2]. Human neutrophil antigen 2 (HNA-2) alloantibodies have been linked to the induction of TRALI and various pulmonary reactions [3–6] while anti-HNA-3 alloantibodies are frequently implicated in severe and fatal TRALI [7]. Animal models have firmly established a pathological role for HNA-2 alloantibodies in TRALI [8,9]. Furthermore, HNA-2 alloantibodies have been implicated in multiple human disorders such as neonatal alloimmune neutropenia, autoimmune neutropenia, drug-induced immune neutropenia, and graft failure following marrow transplantation [10–13]. Accordingly, HNA-2 is among the most important clinical antigens.

**Fig 1:**
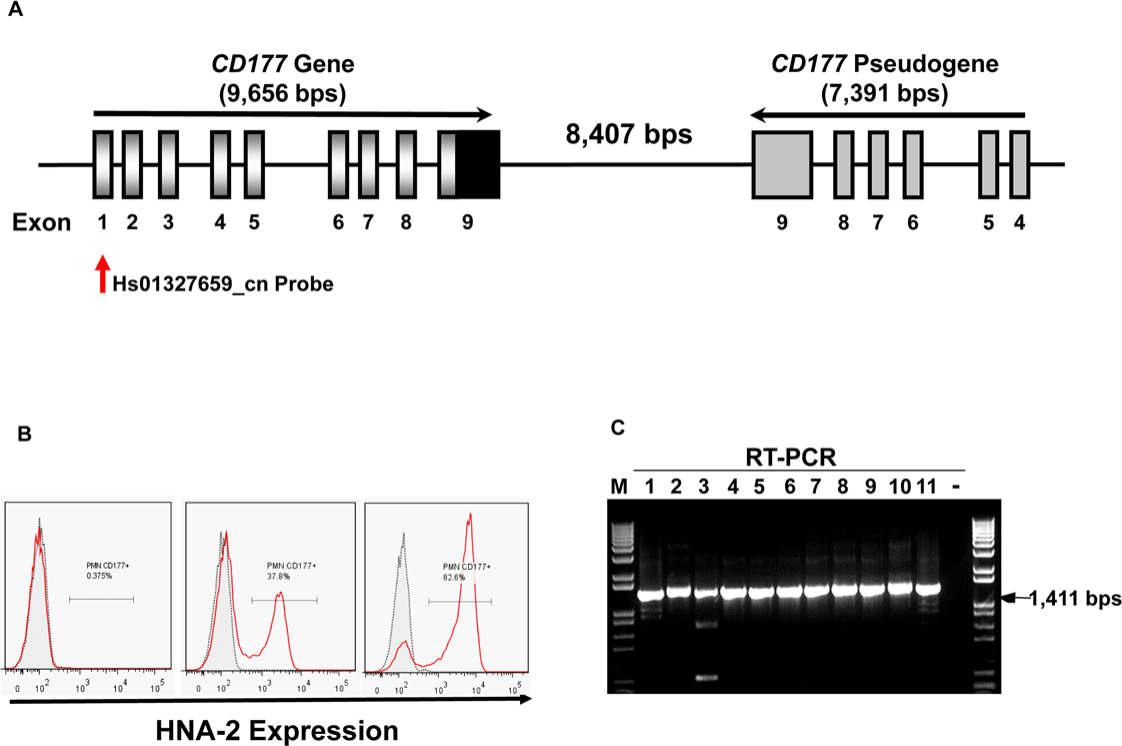
*CD177* gene structure and HNA-2 expression. **A).** Structures of *CD177* gene and pseudogene at chromosome 19q13.31 region. *CD177* gene with nine exons and its pseudogene with six pseudo-exons are separated by 8,407 nucleotides. *CD177* pseudogene is highly homologous to *CD177* between exon 4 and 9. Vertical arrow indicates the CNV assay probe location. **B).** HNA-2 expression varies in different donors. Characteristic light-scatter properties were used to identify neutrophils in flow cytometry. Neutrophils from different donors vary in the percentages of HNA-2 positive cells. Right panel shows the absence of HNA-2 expression on neutrophils from a donor. **C).** Amplification of full-length *CD177* cDNA from HNA-2 deficient donors. All HNA-2 deficiency donors expressed full-length *CD177* mRNA the full-length *CD177* cDNAs (1,411 bps) were detected in RT-PCR.

HNA-2 is heterogeneously expressed on subpopulations of neutrophils and approximately 3–5% Americans do not express HNA-2 [14]. HNA-2 deficient subjects are predisposed to the production of HNA-2 alloantibodies when exposed to the HNA-2 antigen during blood transfusion, pregnancy, and bone marrow transplantation. HNA-2 is encoded by the *CD177* gene that contains nine exons at Chromosome 19q13.31 region, where a *CD177* pseudogene highly homologous to *CD177* between exon 4 and 9 is also located (Fig 1A) [15–17]. The genetic studies of *CD177* were significantly hampered by the presence of *CD177* pseudogene [18,19]. HNA-2 is also known as PRV-1 as *CD177* mRNA is over-expressed in polycythemia rubra vera patients [20]. *CD177* has an open reading frame of 1311 nucleotides that encode 437 amino acids with a signal peptide of 21 residues. HNA-2 (or CD177) is expressed as a GPI-linked receptor with a mature peptide consisting of residue 22 to 408 [15,21]. HNA-2 plays important roles in neutrophil functions and myeloid cell proliferation. The interaction between HNA-2 and PECAM-1 facilitates neutrophil transendothelial migration [22,23]. In addition, HNA-2 is required for the attachment of proteinase 3 (PR3) to neutrophils [24–27], which plays a pivotal role in PR3-ANCA-mediated neutrophil activation [28]. *CD177* mRNA levels are elevated in several conditions associated with increased neutrophil counts [14,29]. Furthermore, elevated levels of neutrophil *CD177* mRNA are associated with increased neutrophil production and quantitation of neutrophil *CD177* mRNA is a diagnostic tool for polycythemia vera [14]. Moreover, the level of HNA-2 expression has been identified as a prognostic biomarker for gastric cancer [30].

The *CD177* non-synonymous coding SNPs (cSNPs) were reported to associate with HNA-2 expression variations, however, the effect of those non-synonymous *CD177* coding SNPs on HNA-2 expression was unknown [18,31,32]. *CD177* mRNA splicing variants were found in two HNA-2 deficient donors but it remains inconclusive whether *CD177* splicing abnormality was actually responsible for HNA-2 deficiency [33]. Therefore, the underlying genetic mechanism of HNA-2 deficiency has remained elusive since the observation of HNA-2 deficiency four decades ago [10]. Elucidation of the molecular genetics and basis of the HNA-2 deficiency is a prerequisite for the use of effective genetic tests in prognosis and diagnosis of HNA-2-related human diseases. In the current study, we demonstrated that a novel nonsense *CD177* coding SNP 829A>T is the primary genetic determinant for HNA-2 deficiency and expression variations in humans.

## Results

### Copy number variations (CNVs) of *CD177* gene

The percentages of neutrophils expressing HNA-2 were heterogeneous among normal healthy blood donors in flow cytometry analysis (Fig 1B). In 294 normal healthy blood donors, the percentage of HNA-2-positive neutrophils ranged from 0.0% to 97.8%. Among 294 blood donors, we have identified 11 donors (or 3.7%) deficient for HNA-2 and the percentage of HNA-2 deficient blood donors is consistent with those previously reported [6,10,34].

Copy number variations (CNVs) are the primary cause of human neutrophil antigen 1 (HNA-1 or FcγRIIIB) deficiency and expression variations [35–38]. To investigate whether *CD177* CNVs are involved in HNA-2 deficiency, we determined *CD177* CNVs using TaqMan CNV assay kit Hs01327659_cn with the probe targeting the unique *CD177* exon 1 region (Fig 1A). Among 294 human subjects, 95.2% (280/294) of subjects were two-copy *CD177* carriers and 4.8% (14/294) were three-copy *CD177* carriers. No human subjects had *CD177* gene deletions among 294 subjects. Notably, all 11 HNA-2 deficient donors identified in the flow cytometry analysis carried two copies of *CD177* gene. In addition, those 11 HNA-2 deficient donors produced full-length *CD177* mRNAs as demonstrated by RT-PCR (Fig 1C). Our data clearly demonstrated that *CD177* gene deletion (or CNVs) and the lack of mRNA expression are not the cause of HNA-2 deficiency.

### Detection of a novel nonsense *CD177* coding SNP (cSNP)

**Fig 2:**
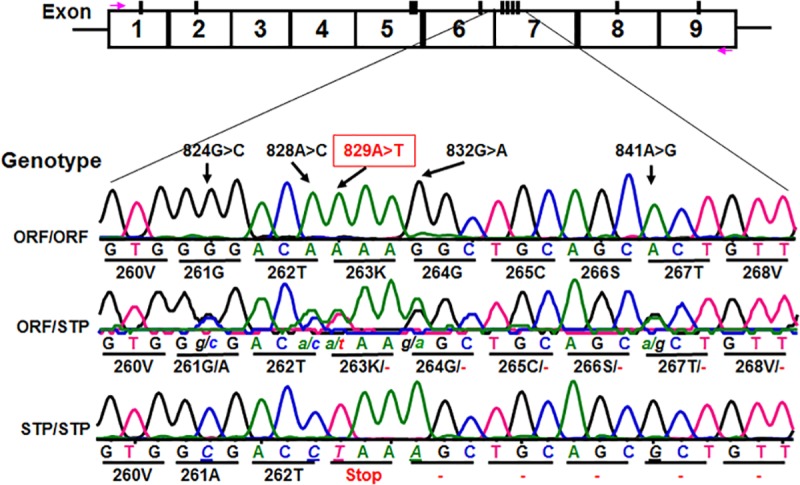
Identification of novel *CD177* coding SNPs and SNP haplotypes. The *CD177* cDNAs from 11 HNA-2 deficiency donors contain five novel cSNPs (824G>C, 828A>C, 829A>T, 832G>A, and 841A>G) that form a previously unidentified haplotype (824C/828C/829T/832A/841G). The *CD177* cSNP 829A>T is a nonsense polymorphism that creates a translation stop codon at amino acid position 263 (Lysine → Stop codon change) in *CD177* open reading frame. The upper row of tracers is the haplotype containing 829A allele (designated as ORF or open reading frame allele) while the lower row tracer shows the haplotype containing 829T allele (designated as STP or stop codon allele). The middle tracer was from a heterozygous donor.

We subsequently determined *CD177* cDNA sequences of all 11 HNA-2 deficient donors along with 119 HNA-2 positive donors. In addition to *CD177* coding SNPs (cSNPs) identified previously, we discovered five novel cSNPs (SNP 824G>C or rs17856827G>C, 828A>C or rs70950396A>C, 829A>T or rs70950396A>T, 832G>A, and 841A>G or rs201266439) (S1 Table), which form two haplotypes (Fig 2). Most importantly, the *CD177* SNP 829A>T is a nonsense polymorphism that creates a translation stop codon at amino acid position 263 (Lysine → Stop codon change) in *CD177* open reading frame. Consequently, those two haplotypes were designated as the open reading frame haplotype (or ORF allele: 824G/828A/829A/832G/841A) and the stop codon haplotype (or STP allele: 824C/828C/829T/832A/841G) (Fig 2). To determine the origin of the novel *CD177* cSNP haplotype, we have also sequenced *CD177* genomic DNA PCR products. Based on genomic DNA sequencing analysis, 72.1% (212/294) of donors were homozygous 829AA donors and the homozygous 829TT donors accounted for 3.1% (9/294) in our study population. The minor allele (829T) frequency is 15.5% (S2 Table). The distribution of SNP 829A>T genotypes was consistent with the Hardy-Weinberg equilibrium in 294 blood donors (χ^2^ = 0.76, *P* = 0.38) (S2 Table).

### Association of the *CD177* SNP 829A>T genotypes with HNA-2 deficiency and expression variations

**Fig 3:**
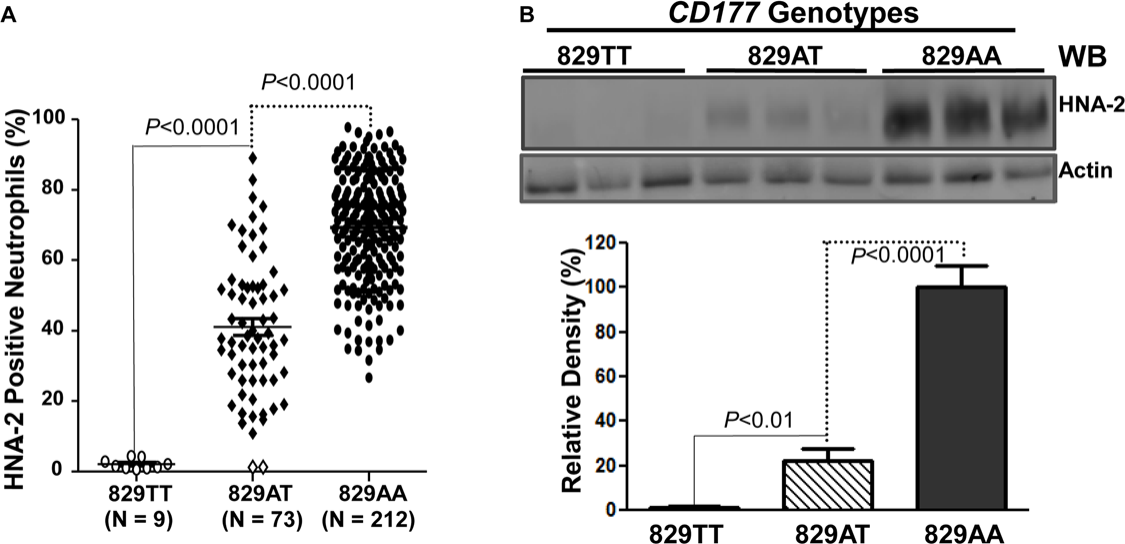
Association of *CD177* SNP 829A>T with HNA-2 expression. **A).** *CD177* genotypes were determined with genomic DNA sequence analysis. HNA-2 expression was examined with flow cytometry analysis. All 829TT homozygous donors (829TT, N = 9) were negative for HNA-2. The percentages of HNA-2 positive neutrophils from heterozygous donors (829AT, N = 73) were significantly (*P* < 0.0001) lower than those from 829AA homozygous donors (829AA, N = 212). Two heterozygous 829AT donors indicated by the empty diamonds in the middle column were also negative for HNA-2 expression. **B**). HNA-2 expression in whole blood leukocytes was assayed in Western blot analyses. Representative blot image is shown (Top panel). No HNA-2 protein could be detected in all 829TT homozygous donors (829TT, N = 9) while heterozygous donors (829AT, N = 9) express significantly less HNA-2 as compared to the 829A homozygous donors (829AA, N = 9) (Lower panel).

To examine whether the *CD177* SNP 829A>T affects HNA-2 expression, the donor genotypes and HNA-2 expressions were statistically analyzed. As shown in Fig 3A, all nine 829TT homozygous donors were negative for HNA-2 expression in flow cytometry analysis. In addition, the percentages of HNA-2 positive neutrophils from 73 heterozygous donors (829AT) were significantly lower than those from 212 homozygous 829AA donors (*P* < 0.0001). Western blot analyses also confirmed the absence of HNA-2 protein in 829TT homozygous donors and significantly less HNA-2 protein being expressed in the 829AT donors when compared to the 829AA homozygous donors (Fig 3B). Our data strongly support the notion that the SNP 829A>T allele is a crucial determinant for HNA-2 deficiency and expression variations. To verify our findings, we recruited an independent cohort containing 102 blood donors, among whom nine HNA-2 deficient donors were identified (S1 Fig). Similar to those of the first cohort, all nine HNA-2 deficient donors in the replication cohort were SNP 829TT homozygotes as demonstrated by sequencing analysis of genomic DNA and cDNA (S2 Fig). Again, the SNP 829A>T genotypes were significantly associated with the percentages of HNA-2 positive neutrophils (S1 Fig) and the HNA-2 protein expression (S3 Fig). Our data confirmed that the SNP 829A>T is a crucial genetic determinant for HNA-2 deficiency and expression variations.

### Identification of a rare mutation in HNA-2 deficiency donors

**Fig 4:**
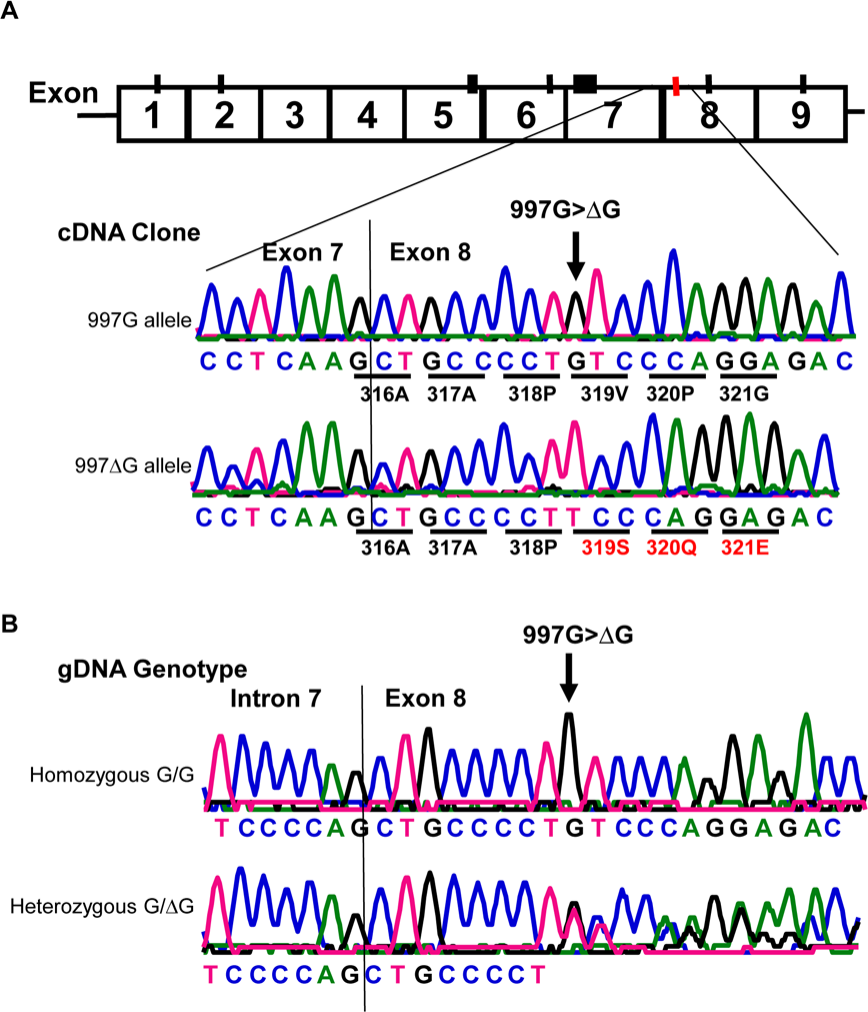
Detection of a rare *CD177* nucleotide deletion mutation. **A).** Sequencing analysis of cDNA clones from two HNA-2 deficient donors who were heterozygous for the SNP 829A>T. The upper tracer is the wild-type (997G allele) *CD177* cDNA clone sequence. The wild-type 997G allele is in linkage disequilibrium with SNP 829T (or STP) allele as demonstrated by the sequence of cDNA clones from two HNA-2 deficient donors. The lower tracer is the sequence of *CD177* cDNA clone with 997G deletion (designated as 997ΔG) that is in linkage disequilibrium with SNP 829A allele (or ORF) in *CD177* cDNA clones. Those two ORF/STP heterozygous donors manifested as HNA-2 deficient phenotype had the combination of one chromosome carrying 997G deletion (997ΔG) and the other carrying the 829T (STP) allele. **B).** Genomic DNA sequence analysis confirmed that the guanidine nucleotide deletion occurs at genomic level. The upper tracer shows the wild-type genomic DNA sequence with the 997G. The lower tracer is the *CD177* genomic sequence of a heterozygous donor with one chromosome containing 997G deletion and the other containing the wild-type.

Similar to all nine homozygous 829TT donors, two 829AT heterozygous donors were also negative for HNA-2 expression (Fig 3A, empty diamonds in the middle column). Analysis of their *CD177* cDNA sequences revealed that both HNA-2 deficient donors who were heterozygous for SNP 829A>T also had a heterozygous deletion of the guanidine nucleotide at nucleotide 997 (997G deletion). To determine haplotypes of the SNP 829A>T and the 997G deletion, we cloned and sequenced cDNA from those two HNA-2 deficient donors. As shown in Fig 4A, two species of *CD177* mRNAs were found in those two donors. The SNP 829T (STP) allele is in the linkage disequilibrium with the wild-type *CD177* 997G allele while the 829A (ORF) allele carries the 997G deletion. Genomic DNA sequence analysis confirmed that the guanidine nucleotide deletion occurs at genomic level (Fig 4B). Our data indicate that the presence of the 829T allele in combination with the deletion mutation at nucleotide 997 on another chromosome could also lead to the HNA-2 expression deficiency in an individual. However, we found that only two out of 294 blood donors carried the 997G deletion mutation at one chromosome with genomic sequencing analysis. Therefore, the allele frequency of the 997G deletion mutation is estimated to be 0.0034 in the study population. In those two 829AT heterozygous donors, the 997G deletion allele was coincidentally paired with the 829T allele, which facilitated the discovery of the rare 997G deletion mutation in the study. We failed to identify any donors with the *CD177* 997G deletion among 102 additional blood donors of the replication cohort, confirming that the 997G deletion is a rare mutation in the population.

### Effect of *CD177* cSNPs on HNA-2 expression and alloantibody binding

**Fig 5:**
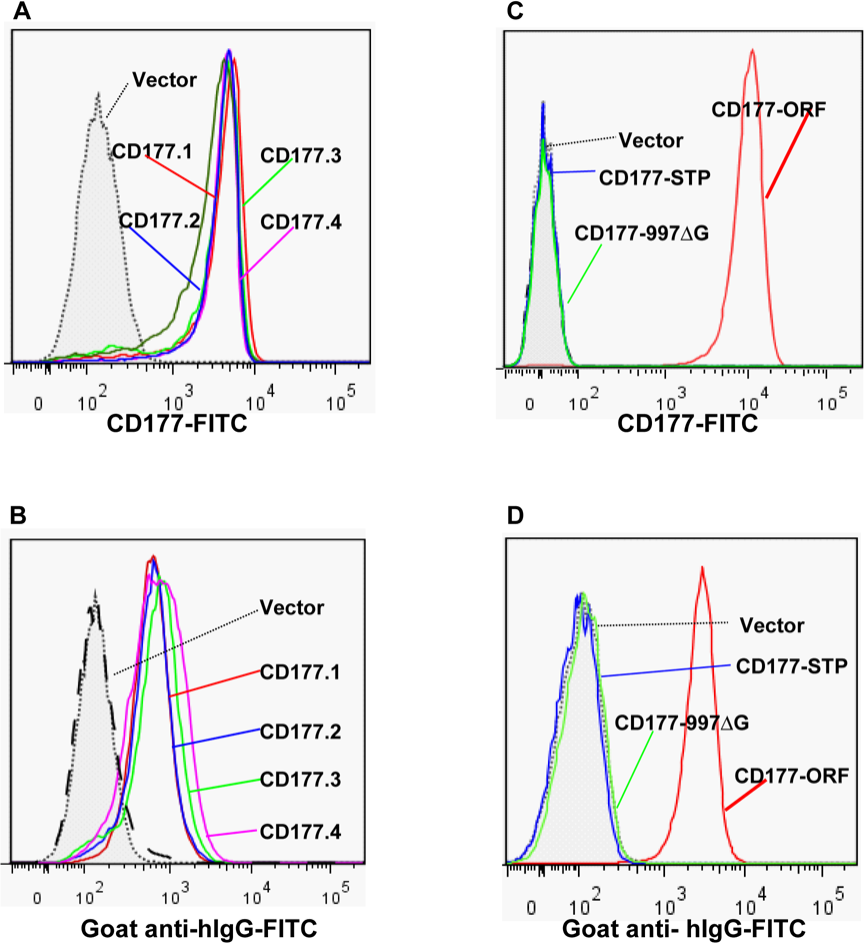
Effect of *CD177* SNPs or mutation on HNA-2 expression and HNA-2 alloantibody binding. **A).** Expression of common *CD177* SNP haplotypes or variants (CD177.1: 31L-204N-205R-348A. CD177.2: 31H-204N-205R-348A. CD177.3: 31L-204D-205M-348A. CD177.4: 31L-204N-205R-348T) on 293 cells. No significant differences of HNA-2 expressions were observed among four *CD177* haplotypes consisting of four common non-synonymous cSNPs (SNP 134A>T, 652A>G, 656G>T, and 1084G>A), which encode the amino acid substitutions for 31His>Leu, 204Asn>Asp, 205Arg>Met, and 348Ala>Thr. **B).** No significant differences were observed in the HNA-2 alloantibody binding to four common *CD177* variants (CD177.1: 31L-204N-205R-348A. CD177.2: 31H-204N-205R-348A. CD177.3: 31L-204D-205M-348A. CD177.4: 31L-204N-205R-348T). **C).** HNA-2 expression was absent in cells stably transfected with the *CD177* expression constructs of the SNP 829T allele (CD177-STP) or 997G deletion mutation (CD177-997ΔG). The SNP 829A allele (or CD177-ORF) serves as the positive control for HNA-2 expression. **D**). Cells stably transfected with the expression constructs containing either SNP 829T (CD177-STP) allele or 997G deletion (CD177-997ΔG) allele failed to react with HNA-2 alloantibodies. The SNP 829A allele (or CD177-ORF) serves as the positive control for HNA-2/CD177 expression. All experiments were repeated at least three times.

Although the genotypes of *CD177* non-synonymous SNPs were reportedly associated with HNA-2 expression variations in several genetic analyses [18,31,32], it is unknown whether those *CD177* cSPNs directly affect HNA-2 expression. To examine the effect of non-synonymous *CD177* cSNPs on HNA-2 expression and on the binding to HNA-2 alloantibodies, we cloned the full-length *CD177* cDNA variants containing common non-conservative cSNPs (SNP 134A>T, 652A>G, 656G>T, and 1084G>A) within the coding region for HNA-2 mature peptide (aa22-408). As shown in Fig 5, there were no significant differences in the expression of HNA-2 (Fig 5A) or in the binding to HNA-2 alloantibodies (Fig 5B) among four *CD177* variants consisting of four non-conservative amino acid substitutions (His31Leu, Asn204Asp, Arg205Met, and Ala348Thr). Our data support the notion that non-synonymous *CD177* cSNPs do not have a direct role in the HNA-2 alloantibody production and expression variations. However, cells transfected with *CD177* variants of either STP haplotype (*CD177*-STP) or 997G deletion (*CD177*-997ΔG) failed to express HNA-2 on cell surface (Fig 5C) and had no reactivity with HNA-2 alloantibodies (Fig 5D). Our data confirmed that either STP allele or 997G deletion mutation will lead to the HNA-2 expression deficiency. To further confirm that the nonsense SNP 829A>T in the STP haplotype is the key factor for HNA-2 expression, we generated a *CD177* expression construct carrying the sole change at SNP 829A>T position. The T substitution at nucleotide position 829 alone led to the absence of HNA-2 expression in transfection experiments (S4 Fig), confirming that the SNP 829A>T is the sole determinant for HNA-2/CD177 expression in the STP haplotype.

## Discussion

The phenomenon of HNA-2 deficiency was observed more than four decades ago [10], however, the underlying genetic mechanism of HNA-2 deficiency has remained unknown. In the current study, we identified five common *CD177* cSNPs (SNP 824G>C, 828A>C, 829A>T, 832G>A, and 841A>G, minor allele frequency = 0.155) in complete linkage disequilibrium. Among five SNPs, the nonsense SNP 829A>T changes the amino acid codon #263 from lysine to a stop codon, which leads to the HNA-2 expression deficiency. Neutrophils from all 829T allele homozygous donors failed to express HNA-2. In addition, the percentages of HNA-2 positive neutrophils from the SNP 829A>T heterozygous donors (ORF/STP) were significantly lower than those from ORF homozygous donors. In vitro, the T substitution at the nucleotide position 829 alone led to HNA-2 expression deficiency in transfection experiments, confirming that the SNP 829A>T is the sole determinant for HNA-2 expression in the STP haplotype. Our study was the first to unravel the genetic mechanism for HNA-2 deficiency, which plays critical roles in human immunological diseases including TRALI, immune neutropenia, and bone marrow graft failure [3–6,10–13]. The delineation of the HNA-2 genetics undoubtedly will enable the development of effective genetic and clinical diagnosis tools in human medicine.

Intriguingly, similar to neutrophils from all homozygous donors of 829T allele, neutrophils from two 829AT heterozygous donors were also negative for HNA-2 expression (Fig 3A). Analysis of cDNA sequences of those two 829AT heterozygous donors deficient for HNA-2 revealed that the 829A allele (or ORF allele) in those two donors had a guanidine deletion at the nucleotide position 997, which leads to the *CD177* reading-frame shift starting from the amino acid codon #319 (Fig 4 **)** and the creation of a stop codon at the amino acid codon #342. The *CD177* 997G deletion also leads to the early termination of HNA-2 peptide translation, similar to the consequence of the 829T allele. Furthermore, the *CD177* variant carrying the nucleotide 997G deletion failed to express HNA-2 on cell surface in the transfection experiments (Fig 5C), confirming the contribution of the 997G deletion mutation to HNA-2 deficiency in those two specific individuals. The *CD177* nucleotide 997G deletion mutation was extremely rare (mutant allele frequency = 0.0034) and was absent in the replication cohort of 102 donors. The coincidental appearance of the 997G deletion allele and the 829T allele in the HNA-2 deficient donors facilitated the discovery of the rare 997G deletion mutation in the study. Therefore, at the presence of 829T allele, the rare *CD177* 997G deletion may also contribute to HNA-2 deficiency. However, the 997G deletion mutation with the allele frequency of 0.0034 will have much less impact on overall HNA-2 deficiency as compared to the SNP 829A>T (the 829T allele frequency was 0.155, S2 Table).

Previous genetic studies suggested that the *CD177* non-synonymous SNPs might affect HNA-2 expression [18,31,32], however, the effect of those *CD177* cSNPs on HNA-2 expression is unclear. In the current study, we carried out transfection experiments to examine whether common non-conservative cSNPs (SNP 134A>T, 652A>G, 656G>T, and 1084G>A) within the HNA-2 mature peptide (aa22-408) affect the HNA-2 expression and the binding of HNA-2 alloantibodies. We found that the expression of HNA-2 and the binding to HNA-2 alloantibodies were not significantly different among those natural *CD177* variants containing non-conservative amino acid substitutions (His31Leu, Asn204Asp, Arg205Met, and Ala348Thr) (Fig 5A and 5B). The expression of HNA-2 in normal neutrophils is also affected by methylations of *CD177* promoter and the *CD177* SNP 42G>C (rs45441892) at the third codon (Pro3Ala) of the HNA-2 signal peptide was associated with methylation levels of CD177 promoter [39]. However, we found no association between the SNP 42G>C genotypes and the percentages of HNA-2 positive neutrophils in our study (ANOVA, *P* = 0.1209, S5 Fig). Taken together, those non-synonymous *CD177* cSNPs do not seem to have a significant effect on HNA-2 deficiency and expression.

The *CD177* mRNA splicing abnormality was previously suggested to be the cause of HNA-2 deficiency as alternatively spliced *CD177* mRNA species were detected in two HNA-2 deficient donors [33]. However, no further evidence was provided to support the alternative splicing hypothesis of HNA-2 deficiency in the report [33]. Alternative mRNA splicing is a physiological process and is an essential mechanism to produce different products from a single human gene [40–42]. It seems unlikely that HNA-2 deficient subjects have an abnormal mRNA splicing machinery as HNA-2 deficient donors appear healthy [6]. We have detected full-length *CD177* mRNAs in all 11 HNA-2 deficient donors in the main study (Fig 1C) and in all nine HNA-2 deficient donors from the replication study. The combination of the alternative spliced *CD177* mRNA isoforms and the regular *CD177* mRNA isoform occurred only in two out of nine SNP 829TT homozygous donors in our replication cohort (S2 Fig). Our data refute the notion that the alternative splicing is a major cause of HNA-2 deficiency.

Although gene deletions or copy number variations (CNVs) are the primary cause for HNA-1 (or FcγRIIIB) deficiency [35–38], we did not find any *CD177* gene deletion in our blood donors. We found that all HNA-2 deficient donors expressed full-length *CD177* mRNAs. We also found that the SNP 829T allele was in complete linkage disequilibrium with SNP 134A, 156G, 593G, 652A, 656G, 671C, 782C, 793C, 824C, 828C, 832A, 841G, 1084G, and 1333G. Our data clearly demonstrated that the gene deletion or the lack of mRNA expression is not responsible for HNA-2 deficiency, in contrast to the HNA-1 deficiency. Interestingly, we found that all heterozygous donors of the SNP 829A>T determined by genomic DNA analysis primarily produced the SNP 829A allele (or ORF allele) mRNA based on their cDNA sequences. The nonsense SNP 829T allele tracer peak barely above the background was typically considered as sequence noise in the cDNA sequence analysis for heterozygous donors. This observation suggests that the *CD177* mRNAs containing the nonsense 829T allele are much less stable than the *CD177* mRNAs containing the common 829A allele within the same donor. This may explain the observation of associations between expression variations and certain *CD177* cSNPs and the inability to discover the SNP 829A>T using the cDNA sequencing strategy in previous studies [31–33].

After transcription, the *CD177* mRNA of the nonsense 829T allele may be quickly degraded by the mechanism of nonsense-mediated mRNA decay [43], which will lead to the low abundance of *CD177* 829T allele mRNA and the dominance of *CD177* 829A allele mRNA in the heterozygous individual. The nonsense-mediated mRNA decay mechanism per se may contribute to the *CD177* mRNA expression deficiency in humans with different diseases, which may explain that the partial HNA-2 peptide was undetectable from those HNA-2 deficient donors in a previous study [44] and in the current study using multiple anti N-terminus of HNA-2 mAbs and HNA-alloantibodies (S6 Fig). Therefore, HNA-2 alloantibodies likely target the whole mature peptide of CD177 in HNA-2 deficient subjects. In heterozygous donors for the SNP 829A>T, only the 829A allele is able to express HNA-2. *CD177* promoter DNA methylation regulates HNA-2 expression under physiologic conditions [39]. Non-selective methylation on the 829A allele alone is sufficient to effectively abrogate the HNA-2 expression in a specific cell during granulopoiesis, which may explain that the percentages of HNA-2 positive granulocytes were significantly lower in the 829A>T heterozygous donors than those in the 829A (or ORF) allele homozygous donors (Figs 3 and S1). Therefore, our data strongly support the concept that the SNP 829A>T is also a primary genetic factor for HNA-2 expression variations in humans.

As an important biomarker, HNA-2 (CD177) is over-expressed in neutrophils from patients with myeloproliferative disorders including polycythemia vera, essential thromobocythemia, idiopathic myelofibrocythemia, and hypereosinophilic syndrome [6,14]. HNA-2 was an indicator of increased erythropoietic activity in thalassemia syndromes as HNA-2 expression was significantly elevated in β-thalassemia patients compared to healthy controls [45]. HNA-2 overexpression may also have a direct role in the pathogenesis of myeloproliferative disorders as HNA-2 enhances cell proliferation in vitro [46,47]. Not surprisingly, the low percentage of HNA-2 positive neutrophil is significantly associated with myelodysplastic syndrome and chronic myelogenous leukemia [48,49], suggesting that the reduced levels of membrane-bound HNA-2 may decrease the proliferation and differentiation potentials of myeloid cells. It is possible that the selection pressure to limit the spread of myeloproliferative disorders during evolution may be an important factor in maintaining the *CD177* nonsense polymorphism in humans. Therefore, the *CD177* SNP 829A>T may be an important genetic risk factor for various myeloproliferative disorders. Approximately 3% of Caucasians, 5% of African Americans, and 1–11% of Japanese are HNA-2 deficient [14]. In the current study, we found that between 3.7% (main cohort) and 8.8% (replication cohort) blood donors (>98% of them were Caucasians from the State of Minnesota) were HNA-2 deficient. Our data indicate that percentages of HNA-2 deficient humans may vary in different regions and be affected by sample sizes.

In summary, the elucidation of the molecular mechanism of HNA-2 deficiency and expression variations fills the critical knowledge gap in the genetics of HNA-2 antigen system. Our findings will enable the development of reliable genetic assays for HNA-2 system and will facilitate the diagnosis and prognosis of HNA-2-associated human disorders.

## Materials and Methods

### Ethics statement

The human study was approved by the Institutional Review Board for Human Use at the University of Minnesota with Study #1301M26461. Memorial Blood Centers (737 Pelham Boulevard, St. Paul, Minnesota 55114) provided healthy donor blood samples without identifications for research purpose as a service and no consent form was provided per the Memorial Blood Centers policy.

### Study subjects

Healthy American blood donors were recruited at the Memorial Blood Center in St. Paul, Minnesota. The age of healthy blood donors ranged from 19 to 84 years-old and >98% of donors in the study were self-declared Caucasians living in the State of Minnesota.

### Evaluation of HNA-2 (CD177) expressions on neutrophils

The expression of HNA-2 and the percentage of HNA-2 positive neutrophils were determined with flow cytometry analysis. Leukocytes stained with either FITC-conjugated anti-CD177 mAb (MEM-166, mIgG1, Thermo Scientific) or mIgG1-FITC isotype control were analyzed on a FACS Canto flow cytometer (BD Biosciences). The FlowJo software (Tree Star Inc.) was used to evaluate flow cytometry data. Characteristic light-scatter properties were used to identify neutrophils in flow cytometry. Using the same criteria as in the literature [31], donors had less than 5% of granulocytes positive for MEM-166 staining in flow cytometry analysis were called as HNA-2 deficient.

### Western blot analysis of HNA-2 protein

Peripheral blood leukocytes (2 × 10^7^ cells) were lysed in PBS containing 1% NP-40 and 1× protease inhibitor cocktail (Roche, Indianapolis, ID) for 1 hr on ice. The total proteins (50 μg) from each donor were used for Western blotting analysis under non-reducing condition with mouse anti-CD177 mAbs and rabbit anti-actin mAb (LI-COR Biosciences, Lincoln, NE). IRDye 800CW-labeled goat anti-mouse and IRDye 600-labeled goat anti-rabbit antibodies were used for imaging analysis with the instrument software on an Odyssey Infrared Imager according to vendor’s instructions (LI-COR Biosciences).

### Nucleic acid isolation

Human genomic DNA was isolated from EDTA anti-coagulated peripheral blood using the Puregene DNA isolation kit (Gentra Systems, Minneapolis, MN) by following the vendor’s instruction. Total RNA was purified from peripheral blood leukocytes using TRIzol total RNA isolation reagent (Invitrogen, Carlsbad, CA).

### Determination of *CD177* CNVs

The CNVs of *CD177* gene were determined using TaqMan Copy Number Assay kit (the probe location of the assay ID Hs01327659_cn is shown in Fig 1A) (Applied Biosystems, Foster City, CA) and RNase P reference assay (Applied Biosystems, Part# 4403326). Duplex quantitative real-time PCR reactions were carried out on an Applied Biosystems 7500 Real-Time PCR System according to the manufacturer’s instructions. All samples were tested in duplicates, and fluorescence signals were normalized to ROX. TaqMan assay quantitative PCR amplification curves were analyzed using 7500 Software on a plate by plate basis, and the CN was assigned from the raw Cq values using CopyCaller software (version 2.0; Applied Biosystems).

### RT-PCR and cDNA sequencing

Five μg of total RNA was used for cDNA synthesis with the SuperScript Preamplification System (Invitrogen). The 1411-bp cDNA fragment covering the entire *CD177* coding region was amplified with RT-PCR using the sense primer (5’-CTGAAAAAGCAGAAAGAGATTACCAGCCACAG-3’) and anti-sense primer (5’-GTCCAAGGCCATTAGGTTATGAGGTCAGA-3’). The PCR reaction was performed with 2 μl of cDNA, 200 nM of each primer, 200 μM of dNTPs, 2.0 mM of MgSO_4_, and 1 U of Platinum *Taq* DNA polymerase High Fidelity (Invitrogen) in a 25 μl reaction volume. Platinum *Taq* High Fidelity DNA polymerase was used as it allows the amplification of complex cDNA or DNA templates with high accuracy and yield. The ABI Veriti 96-well Thermal Cycler was used for the PCR reaction starting with 94°C for 3 min, 35 cycles of denaturing at 94°C for 30 s, annealing at 56°C for 45 s, extension at 68°C for 1 min and 30 s with a final extension at 72°C for 7 min. All the PCR products, treated with ExoSAP-IT (Affymetrix, Santa Clara, CA), were assessed by direct Sanger sequencing on an ABI 3730xl DNA Analyzer with BigDye v3.1 Sequencing kit (Applied Biosystems). *CD177* cDNA was also directly cloned into pCR2.1-TOPO vector (Invitrogen, Carlsbad, CA). Multiple clones containing *CD177* cDNA were selected and subsequently sequenced to confirm CD177 SNPs. Two sense primers and two antisense primers were used to sequence the full-length *CD177* cDNA coding region (sequencing primers are listed in S3 Table). The electropherogram data, aligned by the DNASTAR software (DNAStar, Madison, WI) were used for the identification of gene polymorphisms.

### Genomic DNA sequence analysis of *CD177* gene

Since *CD177* and its pseudogene contain a highly homologous region between exon 4 and 9 (Fig 1A) [16,17], we used the long-template PCR strategy to obtain the *CD177*-specific products for sequence analyses. Long-template PCR was carried out to amplify the *CD177* genomic DNA containing all 9 exons using the sense primer (5’-CTGAAAAAGCAGAAAGAGATTACCAGCCACAG-3’) and antisense primer (5’-GTCCAAGGCCATTAGGTTATGAGGTCAGA-3’). The PCR reaction was performed with 200 ng DNA, 200 nM of each primer, 200 μM of dNTPs, 2.0 mM of MgSO_4_, and 2 U of Platinum *Taq* DNA polymerase High Fidelity (Invitrogen) in a 25 μl reaction volume. The ABI Veriti 96-well Thermal Cycler was used for the PCR reaction starting with 95°C for 3 min; 10 cycles of denaturing at 94°C for 30 s, annealing at 64°C for 30 s, extension at 68°C for 8 min and 30 s; 30 cycles of denaturing at 94°C for 30 s, annealing at 54°C for 30 s, extension at 68°C for 8 min and 30 s; with a final extension at 68°C for 5 min. The *CD177* DNA fragment (8728 base pairs) was sequenced with a primer (5’-TCTTTGCCCCACACTAAACA-3’) on an ABI 3730xl DNA Analyzer with BigDye v3.1 Sequencing kit.

### Generation of HNA-2 expression constructs

The human HNA-2 expression constructs were generated by cloning *Hind* III/*Xba* I-flanked RT-PCR products containing full-length *CD177* coding region (nucleotide position 25 to 1419, GenBank accession number: NM_020406.2) into the eukaryotic expression vector pcDNA3 (Gibco BRL). The *Hind* III/*Xba* I-flanked CD177 cDNA was amplified from the synthesized cDNA of a blood donor using the upper primer 5’-CCC**AAGCTT**ACCAGCCACAGACGGGTCATGAG-3’ and the lower primer 5’-TGC**TCTAGA**GAGGTCAGAGGGAGGTTGAGTGTG-3’. The changes at nucleotide position 134, 652, 656, 824, 828, 829, 832, 841, 997, and 1084 were generated using QuikChange Site-Directed mutagenesis kit (Stratagene, La Jolla, CA) and primer sets listed in the S3 Table.

### Generation of cell lines expressing HNA-2

The 293 cells (human embryonic kidney cell line) from ATCC (ATCC#CRL-1573, Manassas, VA) were maintained in the DMEM medium supplemented with 10% fetal calf serum and L-glutamine (2 mM) in 5% CO_2_. Transfection reactions were carried out in the 100 mm cell culture dishes with the plasmid DNA (20 μg) purified with OMEGA Plasmid Maxi Kit (Omega Bio-Tek, Norcross, GA) and 40 μl of Lipofectamine 2000 reagent (Invitrogen). Transfected cells were cultured in DMEM medium supplemented with 10% fetal calf serum for two days before harvesting the cells for HNA-2 expression or the selection of stable cell lines with the supplement of G418 (final concentration: 1 mg/ml). The polyclonal cells surviving the G418 selection were sorted with Stemcelll EasySep Cell Sorter for equivalent HNA-2 expression. The expression of HNA-2 on the transfected 293 cell lines was determined with FITC-conjugated anti-CD177 mAb as described previously. In addition, five defined HNA-2 alloantibodies from the American Red Cross Neutrophil Serology Laboratory were used to evaluate the binding of HNA-2 to the cell lines expressing *CD177* variants.

### Statistical analysis

The ANOVA and the nonparametric t-test (Mann-Whitney test) were used to determine whether HNA-2 positive cell population sizes and the HNA-2 deficiency are statistically associated with the nonsense *CD177* cSNPs. The χ^2^ test was used to determine whether the observed genotype frequencies are consistent with Hardy-Weinberg equilibrium.

## Supporting Information

S1 Fig Association of *CD177* SNP 829A>T with HNA-2 expression in a replication study. *CD177* genotypes were determined with genomic DNA sequence analysis as described in the main text. HNA-2 expression was examined with flow cytometry analysis. All 829TT homozygous donors (829TT, N = 9) were negative for HNA-2. The percentages of HNA-2 positive neutrophils from heterozygous donors (829AT, N = 27) were significantly (*P* < 0.0001) lower than those from 829AA homozygous donors (829AA, N = 66).
(DOCX)

S2 Fig HNA-2 deficient donor *CD177* cDNA amplification in a replication study and the diagrams of *CD177* splicing isoforms. RT-PCR and DNA sequencing analysis showed that all nine HNA-2 deficient donors were *CD177* SNP 829TT homozygotes (TT-1, TT-2, TT-3, TT-4, TT-5, TT-6, TT-7, TT-8, and TT-9) and expressed full-length *CD177* mRNA (Upper panel). Seven HNA-2 deficient donors (TT-2, TT-3, TT-4, TT-5, TT-6, TT-8, and TT-9) expressed only the regular full-length *CD177* mRNA (*CD177*-1). One HNA-2 deficient donor (TT-7) expressed a mixture of normal full-length *CD177* mRNA (*CD177*-1) and an alternative spliced *CD177* mRNA with an extra exon (alternative exon 6’) (*CD177*-2). Another HNA-2 deficient donor (TT-1) also expressed alternative *CD177* mRNA splicing isoform (*CD177*-3) that contains additional sequences upstream of the exon 4 (exon 4’) (Lower panel). Those two *CD177* alternative splicing isoforms (*CD177*-2 and *CD177*-3) were also detected in two HNA-2 deficient subjects by Kissel et al (Blood, 2002, 99:4231–4233) (Reference 33).
(DOCX)

S3 Fig Western blot analysis of HNA-2 protein expression in subjects of the replication study. HNA-2 protein were undetectable in all nine 829TT homozygous donors (TT-1 to 9) while heterozygous donors (AT-1 to 8, N = 8) express much less HNA-2 compared to the 829AA homozygous donors (AA-1 to 9, N = 9).
(DOCX)

S4 Fig 829A>T substitution disrupts HNA-2 (CD177) expression. HNA-2 (CD177) expression was absent in cells transiently transfected with the *CD177* expression constructs of the SNP 829T allele (CD177-STP), 829T mutation (CD177-829T), or 997G deletion mutation (CD177-997ΔG). The SNP 829A allele (or CD177-ORF) serves as the positive control for HNA-2 expression. The T substitution at nucleotide position 829 alone led to the absence of HNA-2 expression in transfection experiments.
(DOCX)

S5 Fig No association between *CD177* SNP 42C>G genotypes and HNA-2 expression. *CD177* SNP 42C>G genotypes were not associated with HNA-2 expression variations (ANOVA, *P* = 0.1209). 42CC vs 42GG (Mann Whitney t test, *P* = 0.8432). 42CC vs 42CG (Mann Whitney t test, *P* = 0.3126).
(DOCX)

S6 Fig Western blot analyses of HNA-2 protein expression in whole blood leukocytes using alloantibodies and the mAb targeting N-terminus of HNA-2. The mAb CD177 (C5) (Santa Cruz Biotechnology cat# sc-376329, Santa Cruz, CA, USA) targeting residue 27–247 of human CD177 (the mAb data sheet is available at www.scbt.com) was used to detect HNA-2 expression in HNA-2 deficient (829TT) and normal (829AA) donors. If CD177 partial peptide is produced from 829T allele (the reading frame covers amino acid 1–262), CD177 (C5) mAb should be able to detect the peptide with a predicted molecular weight of 30 kD. CD177 (C5) mAb failed to detect any small molecular weight band in 829TT donors (left panel). Additionally, no small molecular weight CD177 could be detected in the leukocyte cell lysates of 829TT donors by HNA-alloantibodies (right panel).
(DOCX)

S1 Table *CD177* coding SNPs in blood donors. ^a^Nucleotide position is based on GenBanK accession # NM_020406.2. ^b^Amino acid position is counted from the ATG start codon. ^c^Among 11 HNA-2 deficient donors, nine donors are homozygous for the rare alleles and two are heterozygous of rare and common allele. None of those SNPs have been previously reported in any publications. The SNP 824G>C was originally assigned to *CD177* pseudogene (CD177P1) with dbSNP # (rs17856827) in dbSNP database. A single dbSNP# rs70950396 was assigned to both SNP 829A>T and 828A>C without the identification of gene (no GeneView is available) in the dbSNP database, which means that the precise location of SNP 829A>T and SNP 828A>C is unknown (the two SNPs could belong to either *CD177* or *CD177* pseudogene). Finally, no frequency data was currently available for the SNP 841A>G with the dbSNP# rs201266439 in the dbSNP database. Therefore, all five highlighted *CD177* SNPs were newly identified and could be considered as novel.
(DOCX)

S2 Table *CD177* SNP 829A>T genotype and allele distributions in blood donors. The distribution of SNP 829A>T genotypes was consistent with the Hardy-Weinberg equilibrium in 294 blood donors (χ^2^ = 0.76, *P* = 0.38). *The expression of HNA-2 was based on results of flow cytometry, Western blot, and transfection analyses.
(DOCX)

S3 Table Mutagenesis and sequencing primers for generation of *CD177* constructs. ^a^Nucleotide position is based on GenBank accession # NM_020406.2. ^b^The target nucleotide mutation is highlighted with bold, italic, and underline.
(DOCX)
