# Correction: Genetic Mechanism of Human Neutrophil Antigen 2 Deficiency and Expression Variations

**DOI:** 10.1371/journal.pgen.1005360

**Published:** 2015-06-26

**Authors:** The PLOS Genetics Staff

There is an error in the Funding section. The correct funding information is as follows: The research was supported by National Institute of Health grant R21HL117652 and grants from University of Minnesota Academic Health Center. The funders had no role in study design, data collection and analysis, decision to publish, or preparation of the manuscript. The publisher apologizes for the error.
