# Supplementary figures and images for: Genetic Mechanism of Human Neutrophil Antigen 2 Deficiency and Expression Variations
Source: PLoS Genet. 2015 May 29;11(5):e1005255. doi: 10.1371/journal.pgen.1005255 (PMC4449163; doi:10.1371/journal.pgen.1005255)

**Supplemental Figure S1.** Association of *CD177* SNP 829A>T with HNA-2 expression in a replication study


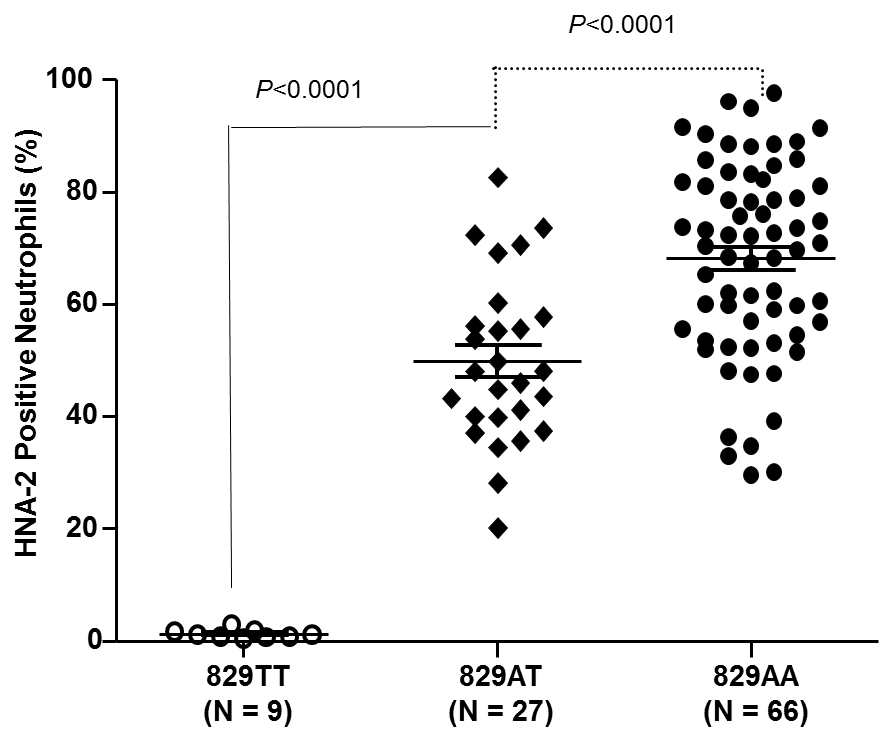

Supplement: S1 Fig — CD177 genotypes were determined with genomic DNA sequence analysis as described in the main text. HNA-2 expression was examined with flow cytometry analysis. All 829TT homozygous donors (829TT, N = 9) were negative for HNA-2. The percentages of HNA-2 positive neutrophils from heterozygous donors (829AT, N = 27) were significantly (P < 0.0001) lower than those from 829AA homozygous donors (829AA, N = 66). (DOCX) [file pgen.1005255.s001.docx]

**Supplemental Figure S5.** No association between *CD177* SNP 42C>G genotypes and HNA-2 expression


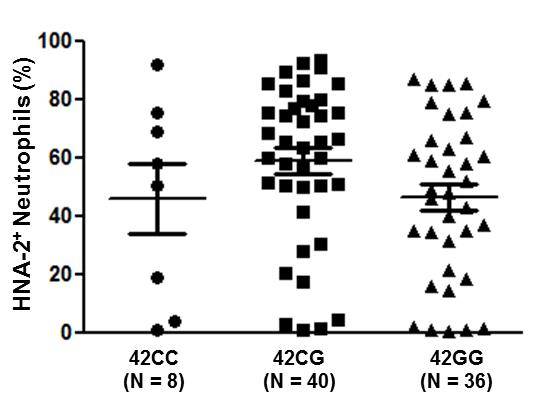

Supplement: S5 Fig — CD177 SNP 42C>G genotypes were not associated with HNA-2 expression variations (ANOVA, P = 0.1209). 42CC vs 42GG (Mann Whitney t test, P = 0.8432). 42CC vs 42CG (Mann Whitney t test, P = 0.3126). (DOCX) [file pgen.1005255.s005.docx]
