# Supplementary material for: Genetic Mechanism of Human Neutrophil Antigen 2 Deficiency and Expression Variations
Source: PLoS Genet. 2015 May 29;11(5):e1005255. doi: 10.1371/journal.pgen.1005255 (PMC4449163; doi:10.1371/journal.pgen.1005255)

**Supplemental Figure S2.** HNA-2 deficient donor *CD177* cDNA amplification in a replication study and the diagrams of *CD177* splicing isoforms


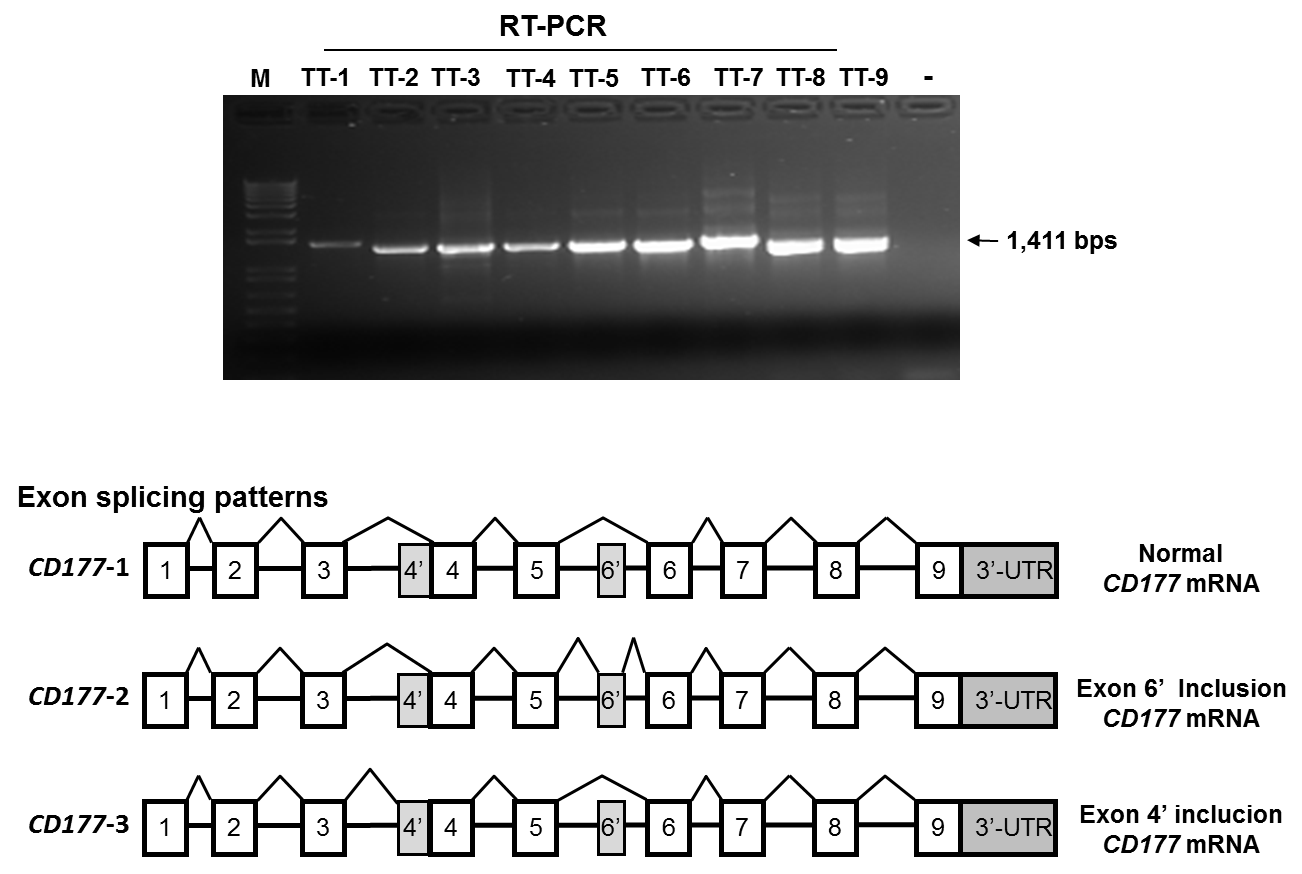

Supplement: S2 Fig — RT-PCR and DNA sequencing analysis showed that all nine HNA-2 deficient donors were CD177 SNP 829TT homozygotes (TT-1, TT-2, TT-3, TT-4, TT-5, TT-6, TT-7, TT-8, and TT-9) and expressed full-length CD177 mRNA (Upper panel). Seven HNA-2 deficient donors (TT-2, TT-3, TT-4, TT-5, TT-6, TT-8, and TT-9) expressed only the regular full-length CD177 mRNA (CD177-1). One HNA-2 deficient donor (TT-7) expressed a mixture of normal full-length CD177 mRNA (CD177-1) and an alternative spliced CD177 mRNA with an extra exon (alternative exon 6’) (CD177-2). Another HNA-2 deficient donor (TT-1) also expressed alternative CD177 mRNA splicing isoform (CD177-3) that contains additional sequences upstream of the exon 4 (exon 4’) (Lower panel). Those two CD177 alternative splicing isoforms (CD177-2 and CD177-3) were also detected in two HNA-2 deficient subjects by Kissel et al (Blood, 2002, 99:4231–4233) (Reference 33). (DOCX) [file pgen.1005255.s002.docx]
