# Supplementary material for: Genetic Mechanism of Human Neutrophil Antigen 2 Deficiency and Expression Variations
Source: PLoS Genet. 2015 May 29;11(5):e1005255. doi: 10.1371/journal.pgen.1005255 (PMC4449163; doi:10.1371/journal.pgen.1005255)

**Supplemental Figure S3.** Western blot analysis of HNA-2 protein expression in subjects of the replication study.


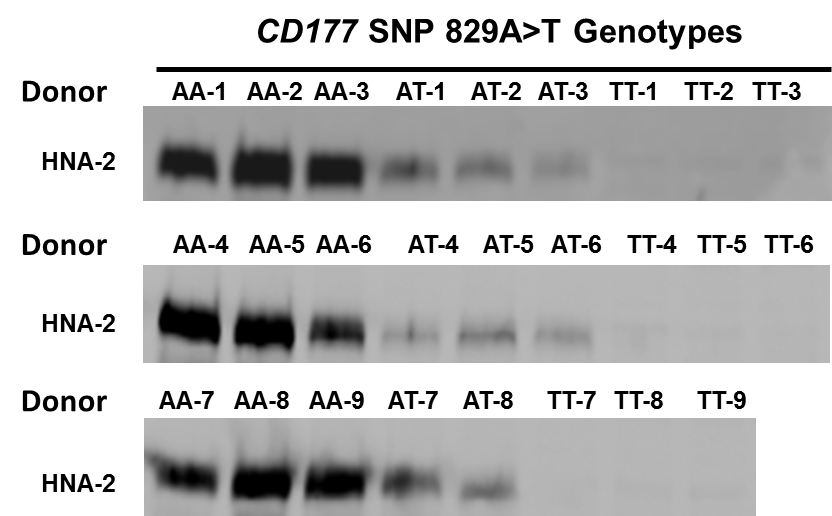

Supplement: S3 Fig — HNA-2 protein were undetectable in all nine 829TT homozygous donors (TT-1 to 9) while heterozygous donors (AT-1 to 8, N = 8) express much less HNA-2 compared to the 829AA homozygous donors (AA-1 to 9, N = 9). (DOCX) [file pgen.1005255.s003.docx]
