# Supplementary material for: Genetic Mechanism of Human Neutrophil Antigen 2 Deficiency and Expression Variations
Source: PLoS Genet. 2015 May 29;11(5):e1005255. doi: 10.1371/journal.pgen.1005255 (PMC4449163; doi:10.1371/journal.pgen.1005255)
Supplement: S4 Fig — HNA-2 (CD177) expression was absent in cells transiently transfected with the CD177 expression constructs of the SNP 829T allele (CD177-STP), 829T mutation (CD177-829T), or 997G deletion mutation (CD177-997ΔG). The SNP 829A allele (or CD177-ORF) serves as the positive control for HNA-2 expression. The T substitution at nucleotide position 829 alone led to the absence of HNA-2 expression in transfection experiments. (DOCX) [file pgen.1005255.s004.docx]

**Supplemental Figure S4.** 829A>T substitution disrupts HNA-2 (CD177) expression
