# Supplementary material for: Genetic Mechanism of Human Neutrophil Antigen 2 Deficiency and Expression Variations
Source: PLoS Genet. 2015 May 29;11(5):e1005255. doi: 10.1371/journal.pgen.1005255 (PMC4449163; doi:10.1371/journal.pgen.1005255)

**Supplemental Figure S6.** Western blot analyses of HNA-2 protein expression in whole blood leukocytes using alloantibodies and the mAb targeting N-terminus of HNA-2.


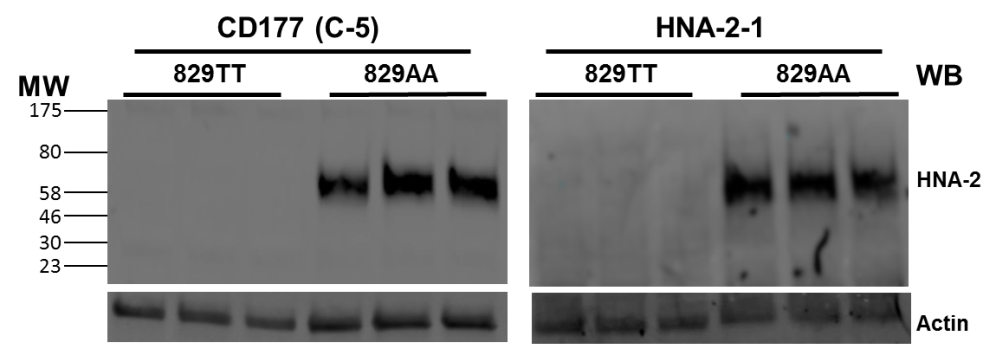

Supplement: S6 Fig — The mAb CD177 (C5) (Santa Cruz Biotechnology cat# sc-376329, Santa Cruz, CA, USA) targeting residue 27–247 of human CD177 (the mAb data sheet is available at www.scbt.com) was used to detect HNA-2 expression in HNA-2 deficient (829TT) and normal (829AA) donors. If CD177 partial peptide is produced from 829T allele (the reading frame covers amino acid 1–262), CD177 (C5) mAb should be able to detect the peptide with a predicted molecular weight of 30 kD. CD177 (C5) mAb failed to detect any small molecular weight band in 829TT donors (left panel). Additionally, no small molecular weight CD177 could be detected in the leukocyte cell lysates of 829TT donors by HNA-alloantibodies (right panel). (DOCX) [file pgen.1005255.s006.docx]
