# Supplementary material for: Genetic Mechanism of Human Neutrophil Antigen 2 Deficiency and Expression Variations
Source: PLoS Genet. 2015 May 29;11(5):e1005255. doi: 10.1371/journal.pgen.1005255 (PMC4449163; doi:10.1371/journal.pgen.1005255)
Supplement: S1 Table — aNucleotide position is based on GenBanK accession # NM_020406.2. bAmino acid position is counted from the ATG start codon. cAmong 11 HNA-2 deficient donors, nine donors are homozygous for the rare alleles and two are heterozygous of rare and common allele. None of those SNPs have been previously reported in any publications. The SNP 824G>C was originally assigned to CD177 pseudogene (CD177P1) with dbSNP # (rs17856827) in dbSNP database. A single dbSNP# rs70950396 was assigned to both SNP 829A>T and 828A>C without the identification of gene (no GeneView is available) in the dbSNP database, which means that the precise location of SNP 829A>T and SNP 828A>C is unknown (the two SNPs could belong to either CD177 or CD177 pseudogene). Finally, no frequency data was currently available for the SNP 841A>G with the dbSNP# rs201266439 in the dbSNP database. Therefore, all five highlighted CD177 SNPs were newly identified and could be considered as novel. (DOCX) [file pgen.1005255.s007.docx]

Supplemental Table S1. *CD177* coding SNPs in blood donors

| Exon | Nucleotide Substitution^a^ | Amino Acid Substitution^b^ | dbSNP# | Donors with minor allele |
| --- | --- | --- | --- | --- |
| 2 | 134A>T | 31His>Leu | rs45553433 | 12/130 |
| 2 | 156G>A | 38Leu>Leu | rs45571738 | 15/130 |
| 5 | 593G>T | 184Gly>Val | rs71337594 | 6/130 |
| 5 | 652A>G | 204Asn>Asp | rs199668750 | 7/130 |
| 5 | 656G>T | 205Arg>Met | rs200662237 | 8/130 |
| 6 | 671C>T | 210Thr>Ile | rs182368720 | 1/130 |
| 6 | 782C>T | 247Thr>Met | rs148509022 | 3/130 |
| 6 | 793C>A | 251Leu>Ile | rs10425835 | 80/130 |
| **7** | **824G>C** | **261Gly>Ala** | **rs17856827** | **11/130**^c^ |
| **7** | **828A>C** | **262Thr>Thr** | **rs70950396** | **11/130**^c^ |
| **7** | **829A>T** | **263Lys>Stop** | **rs70950396** | **11/130**^c^ |
| **7** | **832G>A** | **264Gly>Ser** | **unknown** | **11/130**^c^ |
| **7** | **841A>G** | **267Thr>Ala** | **rs201266439** | **11/130**^c^ |
| 8 | 1084G>A | 348Ala>Thr | rs61625631 | 48/130 |
| 9 | 1333G>A | 431Gly>Arg | rs78718189 | 21/130 |
