# Supplementary material for: Genetic Mechanism of Human Neutrophil Antigen 2 Deficiency and Expression Variations
Source: PLoS Genet. 2015 May 29;11(5):e1005255. doi: 10.1371/journal.pgen.1005255 (PMC4449163; doi:10.1371/journal.pgen.1005255)
Supplement: S2 Table — The distribution of SNP 829A>T genotypes was consistent with the Hardy-Weinberg equilibrium in 294 blood donors (χ2 = 0.76, P = 0.38). *The expression of HNA-2 was based on results of flow cytometry, Western blot, and transfection analyses. (DOCX) [file pgen.1005255.s008.docx]

Supplemental Table S2. *CD177* SNP 829A>T genotype and allele distributions in blood donors

|  | **Human Subjects (N = 294)** | **Expression of HNA-2*** |
| --- | --- | --- |
| **Genotype** |  |  |
| AA (%) | 212 (72.1%) | High |
| AT (%) | 73 (24.8%) | Low - Medium |
| TT (%) | 9 (3.1%) | Negative |
| **Allele** |  |  |
| A (%) | 497 (84.5) | Yes |
| T (%) | 91 (15.5) | No |
