# Supplementary material for: Genetic Mechanism of Human Neutrophil Antigen 2 Deficiency and Expression Variations
Source: PLoS Genet. 2015 May 29;11(5):e1005255. doi: 10.1371/journal.pgen.1005255 (PMC4449163; doi:10.1371/journal.pgen.1005255)
Supplement: S3 Table — aNucleotide position is based on GenBank accession # NM_020406.2. bThe target nucleotide mutation is highlighted with bold, italic, and underline. (DOCX) [file pgen.1005255.s009.docx]

Supplemental Table S3. Mutagenesis and sequencing primers for generation of *CD177* constructs

| **Nucleotide Change^a^** | **Primer Sequences (5’→3’)^b^** |
| --- | --- |
| 134A>T | F: GACAGTTCAGC***T^134^***TGTTGTGA  R: TCGACACA***A^134^***GCTGAACTGTC |
| 652A/656G>652G/656T | F: ACTGAGAACTGC***G^652^***ATA***T^656^***GAAAGATTTTCTG  R: CAGAAAATCTTTC***A***^656^TAT***C***^652^GCAGTTCTCAGT |
| 1084G>A | F: CCCAGGGGC***G^1084^***CCACTCATTGT  R: ACAATGAGTGG***C^1084^***GCCCCTGG |
| 824G/828A/829A/832G/841A>  824C/828C/829T/832A/841G | F: ACCCTGGTGG***C^824^***GAC***C^829^T^829^***AA***A^832^***GCTGCAGC***G^841^***CTGTTGGGGCT  R: AGCCCCAACAG***C^841^***GCTGCAGC***T^832^***TT***A^829^G^828^***GTC***G^824^***CCACCAGGGT |
| 829A>T | F: GTGGGGACA***T^829^***AAGGCTGCAGCA  R: TGCTGCAGCCTT***A^82^***^9^TGTCCCCAC |
| 997G>997ΔG | F: CCTCAAGCTGCCCCT***-^997^***TCCCAGGAGACCGG  R: CCGGTCTCCTGGGA***-^997^***AGGGGCAGCTTGAGG |
| Sequencing primers for cDNA insert | F1: CCT TGA GGT GCC CAG TCT GCT T  F2: ACC GGC AGT GTC CTA CCT GTG T  R1: AAG CAG ACT GGG CAC CTC AAG G  R2: ACA CAG GTA GGA CAC TGC CGG T |
